# Free vs. Local Tissue Transfer and Reconstruction in Pediatric Head and Neck Cancer Patients: A Comparable Complication Outcome Review

**DOI:** 10.3390/medicina61081477

**Published:** 2025-08-18

**Authors:** Valeria Mejia, Asli Pekcan, Melanie Bakovic, Raina Kushal Patel, Marvee Turk, Idean Roohani, Pasha Shakoori, Mark Urata, Jeffrey A. Hammoudeh

**Affiliations:** 1 Division of Plastic and Maxillofacial Surgery, Children’s Hospital Los Angeles, Los Angeles, CA 90027, USA; mejiaval@usc.edu (V.M.); apekcan@usc.edu (A.P.); mbakovic@chla.usc.edu (M.B.); rp1677@mynsu.nova.edu (R.K.P.); murata@chla.usc.edu (M.U.); 2 Division of Plastic and Reconstructive Surgery, Keck School of Medicine, Los Angeles, CA 90033, USA; marvee.turk@med.usc.edu (M.T.); iroohani@usc.edu (I.R.); 3 School of Medicine and Health Sciences, The George Washington University, Washington, DC 20037, USA; 4 Dr. Kiran C. Patel College of Allopathic Medicine, Nova Southeastern University, Fort Lauderdale, FL 33314, USA; 5 Division of Oral and Maxillofacial Surgery, Keck School of Medicine, Los Angeles, CA 90033, USA; 6 Herman Ostrow School of Dentistry, University of Southern California, Los Angeles, CA 90089, USA

**Keywords:** pediatric head and neck cancer, free flap, local flap, microsurgery, wound complications, surgery outcomes, reconstruction, osteosarcoma, rhabdomyosarcoma

## Abstract

*Background and Objectives*: Reconstructive outcomes following head and neck (H&N) cancer resection in pediatric patients remain understudied, particularly regarding the comparative efficacy of free versus local tissue transfer. *Materials and Methods*: A retrospective review was conducted on pediatric patients undergoing malignant H&N tumor resection at a tertiary center from 2007 to 2024. Patients were stratified by reconstruction type (free vs. local flap), and outcomes assessed included flap failure, wound complications, revision rates, operative time, hospital stay, and 30-day readmission. *Results*: A total of 41 patients (mean age: 10.6 years) met inclusion criteria; 18 underwent free flaps and 23 received local flaps. Common diagnoses included osteosarcoma (21.9%) and rhabdomyosarcoma (12.2%). Anterolateral thigh (44.4%) and fibula (33.3%) were the most common free flaps; temporalis (21.7%) and pectoralis (13.0%) were common local flaps. Flap survival was high in both groups (94.4% vs. 100%). However, local flaps had significantly higher rates of hardware exposure (34.7% vs. 5.5%, *p* = 0.025) and wound dehiscence (39.1% vs. 5.5%, *p* = 0.045). Free flaps were associated with longer operative times (10.3 vs. 6.5 h, *p* = 0.011) and hospital stays (29.1 vs. 13.9 days, *p* = 0.036). *Conclusions*: While both approaches achieved high flap survival, free flaps may offer more durable reconstruction and reduce wound-related complications in complex pediatric H&N oncologic cases.

## 1. Introduction

Pediatric head and neck (H&N) cancers are rare, with an estimated incidence of 20 cases per 100,000 children annually [1]. Surgical resection often results in complex, multidimensional defects that pose unique reconstructive challenges not typically encountered in adults. These include smaller vessel caliber, limited donor tissue availability, and the need to accommodate ongoing craniofacial growth [2,3]. In addition, children are particularly vulnerable to the adverse effects of adjuvant therapies such as radiation and chemotherapy, which can impair healing and compromise soft tissue quality [2,4].

While reconstructive strategies in pediatric H&N surgery generally mirror those in adults—adhering to the principles of the reconstructive ladder—additional age-specific considerations must guide decision-making. Primary closure is preferred when feasible, but the extent of many pediatric resections frequently necessitates flap-based reconstruction. Local and regional flaps, such as the temporalis, pectoralis major, or trapezius, offer reliable options for moderate soft tissue defects. For larger or composite defects—especially those involving osseous reconstruction, irradiated fields, or dynamic facial subunits—free tissue transfer provides more durable, vascularized coverage [3,4,5]. However, flap selection in children must also account for growth potential, long-term functional outcomes, and donor site morbidity. Despite technical complexity, microsurgical reconstruction has demonstrated high success rates in pediatric populations when performed by experienced teams [5,6,7,8,9,10,11].

In clinical practice, both free and local flaps remain viable approaches, with selection often influenced by anatomical complexity, comorbidities, oncologic therapy, and institutional resources [12]. While free flaps are increasingly favored for their versatility and long-term durability, local flaps are often chosen in younger children or when microsurgical reconstruction is contraindicated. As such, optimizing flap selection in this high-risk population requires data-driven insights into the comparative outcomes of each technique.

Despite a growing body of literature supporting microsurgical reconstruction in children [13,14,15,16,17], few studies have focused specifically on pediatric patients undergoing oncologic H&N resection [18,19,20,21]. Even fewer have directly compared outcomes between free and local flaps in this context [18,20,22]. This lack of comparative data limits the development of evidence-based reconstructive algorithms in pediatric oncology, particularly for patients facing compounding risks from chemotherapy, radiation, malnutrition, and immunosuppression [23,24,25].

In light of the limited comparative data available, our study evaluates outcomes following malignant H&N tumor resection in children, comparing free versus local flap techniques. By analyzing a single-institution cohort, we aim to provide clinical insights that inform flap selection and optimize long-term outcomes in pediatric H&N cancer reconstruction.

## 2. Materials and Methods

This retrospective study reviewed pediatric patients who underwent reconstruction by the Plastic and Reconstructive Surgery (PRS) team for malignant head and neck defect coverage at a single tertiary children’s hospital between 2007 and 2024. The study was approved by the Institutional Review Board (IRB CHLA-24-00017) and conducted in accordance with the Declaration of Helsinki.

Patients were eligible for inclusion if they were 20 years of age or younger at the time of surgery, had a histologically confirmed malignant head and neck tumor, underwent oncologic resection followed by immediate reconstruction with either free tissue transfer or local/regional flap closure, and had a minimum of six months of postoperative follow-up. Patients were excluded if they were older than 20 years, had benign tumors, underwent reconstruction for non-oncologic indications such as trauma or facial reanimation (e.g., free gracilis flap for facial palsy), or lacked sufficient follow-up data.

At this institution, head and neck malignancies are typically resected by the PRS team, while intracranial tumors are primarily managed by the neurosurgery team, with reconstructive involvement by PRS only when soft tissue coverage is needed. Microsurgical reconstruction was performed in cases involving large, complex, or composite defects requiring vascularized bone and/or soft tissue. All flap reconstructions were performed by two senior attending surgeons with over 20 years of experience.

Patient demographics, tumor histology, reconstructive technique (free vs. local flap), and specific flap type were extracted from operative reports and electronic medical records. Postoperative course and complications were assessed through inpatient and outpatient notes, with late complications, donor site outcomes, and functional or growth-related concerns reviewed through longitudinal follow-up.

Primary outcomes included surgical complications such as flap failure, wound dehiscence, infection, hematoma, and hardware exposure. Postoperative complications were categorized using a clinically relevant classification system which distinguishes between major and minor complications based on the need for surgical intervention, hospitalization, or intravenous therapy. Major complications were defined as events requiring operative management (e.g., hematoma evacuation, flap revision), hospital readmission, or systemic treatment such as intravenous antibiotics or anticoagulation. Minor complications included those manageable with observation, oral antibiotics, or conservative outpatient care without the need for hospitalization or further surgery [26]. Secondary outcomes included operative time, anesthesia duration, hospital length of stay, 30-day readmission, donor site morbidity (including infection, contracture, or need for surgical revision), and perioperative mortality.

Statistical analyses were performed using Stata version 18.5 (StataCorp, College Station, TX, USA). Normality of continuous variables was assessed using histograms and the Shapiro–Wilk test. Continuous data were analyzed using independent samples *t*-tests, while categorical variables were compared using Chi-squared tests. A *p*-value of less than 0.05 was considered statistically significant. The total number of patients who met inclusion criteria and underwent reconstruction is reported in the Results section.

## 3. Results

### 3.1. Patient Selection and Cohort Summary

**Figure 1:**
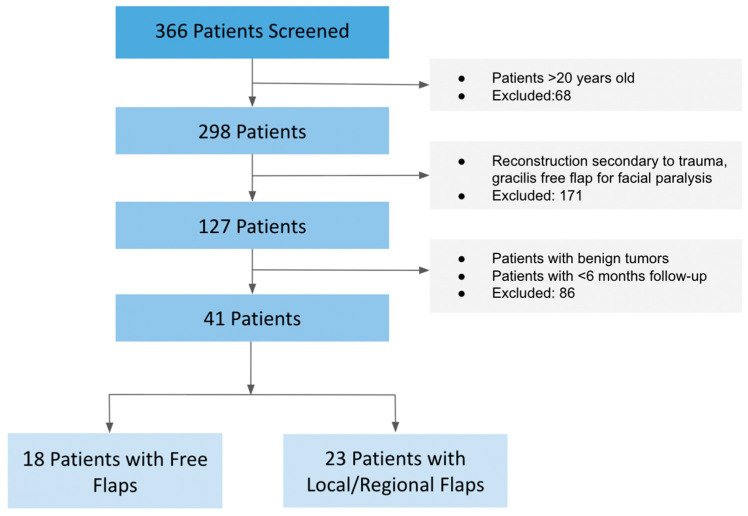
Patient selection and flap cohort distribution Patient screening and inclusion flowchart. Of 366 screened patients with free or local/regional tissue transfer, 41 met inclusion criteria after exclusions for trauma, benign tumors, facial paralysis flaps, age > 20 years, or follow-up < 6 months. Among them, 18 underwent free tissue transfer, and 23 underwent local tissue transfer.

Between 2007 and 2024, a total of 366 pediatric patients underwent head and neck reconstruction with either free or local tissue transfer at our institution. Of these, 325 were excluded due to benign pathology, trauma, facial reanimation procedures (e.g., gracilis flaps), or insufficient follow-up (<6 months). The remaining 41 patients (11.9%) met inclusion criteria, having undergone oncologic resection of malignant tumors followed by immediate reconstruction. Among these, 18 patients (43.9%) underwent free flap reconstruction, and 23 (56.1%) underwent local flap reconstruction (Figure 1).

### 3.2. Demographics and Tumor Characteristics

The mean age at the time of surgery was 10.6 years (range: 6 months to 20 years). Twenty-one patients (51.2%) were male, and 20 (48.8%) were female. The mean follow-up duration was 2.3 ± 2.9 years, with no significant difference between the local flap (2.7 ± 3.8 years) and free flap (1.8 ± 1.8 years) cohorts (*p* = 0.788). Sarcomas accounted for the majority of diagnoses (65.9%). The most common tumor types included osteosarcoma (21.9%), rhabdomyosarcoma (12.2%), and spindle cell sarcoma (14.6%). Preoperative radiation was administered in 65.9% of patients, and all patients received neoadjuvant chemotherapy. Tumor type and treatment characteristics were similar between cohorts (Table 1).

### 3.3. Flap Types and Reconstruction Methods

Among the 18 patients who underwent free flap reconstruction, the most commonly used flaps were anterolateral thigh (ALT) (44.4%), fibula (33.3%), gracilis (16.7%), and rectus abdominis (5.6%). For local flap reconstruction (*n* = 23), the temporalis muscle flap was most frequently used (21.7%), followed by the pectoralis major and trapezius flaps (13.0% each) (Table 2).

### 3.4. Flap Survival and Surgical Complications

The overall flap survival rate was 97.6%. Free flaps had a survival rate of 94.4%, with one flap failure due to venous congestion, while all local flaps survived. Major revisions were required in 21 cases (51.2%), affecting 56.5% of local flaps and 44.4% of free flaps (*p* = 0.326). Hardware exposure occurred significantly more frequently in the local flap cohort (34.7%) than the free flap cohort (5.5%) (*p* = 0.025). Wound dehiscence was also more common in local flaps (39.1% vs. 5.5%, *p* = 0.045). Other complications, including partial flap necrosis and hematoma, showed no statistically significant differences between groups (Table 3). Free flap failure occurred in 3.7% of patients with prior radiation (1/27) and 0.0% of those without (0/14) (*p* = 0.466).

### 3.5. Infectious Complications

Infectious complications were observed in 17.1% of patients. Among free flap patients, infections included soft tissue infections (11.1%) and abscesses (5.6%). No cerebrospinal fluid (CSF) infections occurred in this group. In contrast, 17.4% of local flap patients developed infections, including soft tissue infections (8.7%), abscesses (4.3%), and CSF infection (4.3%).

### 3.6. Operative and Hospitalization Metrics

The average operative time was 8.2 ± 5.1 h, with free flap reconstructions requiring significantly more time than local flaps (10.3 ± 0.8 vs. 6.5 ± 1.2 h, *p* = 0.011). Anesthesia times were also longer for free flaps (12.5 ± 4.1 vs. 7.9 ± 5.9 h, *p* = 0.015). The mean length of hospitalization was significantly longer for the free flap cohort (29.1 ± 7.9 days) than the local flap cohort (13.9 ± 3.5 days, *p* = 0.036). Thirty-day readmission occurred in 32.5% of patients, with a non-significant trend toward higher rates in the local flap group (43.5% vs. 17.6%, *p* = 0.085) (Table 4).

### 3.7. Donor Site Morbidity and Mortality

Donor site morbidity was low overall (2.4%), with one case in the free flap group and none in the local flap group (*p* = 0.252). The average number of total flap reoperations was 1.4 ± 1.9, with no significant difference between groups. Mortality occurred in 26.8% of patients, with similar rates between local (26.1%) and free flap (27.8%) cohorts (*p* = 0.903). The average time from tissue transfer to death was shorter in the local flap group (1.2 ± 0.6 years) compared to the free flap group (2.3 ± 1.0 years), although not statistically significant (*p* = 0.235) (Table 5).

## 4. Discussion

Reconstruction following malignant tumor resection in pediatric patients requires nuanced decision-making to balance defect coverage, functional preservation, long-term growth, and complication risk. This study presents one of the few comparative analyses of local versus free flap reconstruction in children with head and neck malignancies. Our findings demonstrate that both approaches yielded high flap survival (97.6%), but local flaps were associated with significantly higher rates of wound dehiscence and hardware exposure, complications that can increase the need for revision surgery in children.

Historically, concerns over technical complexity and vessel size have limited the adoption of free tissue transfer in the pediatric population. However, mounting evidence over the past decade has demonstrated the safety, reliability, and long-term functional benefits of microsurgical reconstruction in children [27,28,29,30]. Starnes-Roubaud et al. reported outcomes in 109 pediatric patients undergoing oncologic free flap reconstruction, with a flap survival rate of 95.4% even among patients receiving preoperative chemoradiation [27]. Akçal et al. described a 93.7% success rate among 30 children treated with free flaps, including those with head and neck defects, confirming its utility across a range of clinical scenarios [28]. Zavala et al. demonstrated excellent functional results with fibula free flaps in pediatric mandibular reconstruction, including high rates of solid food intake and preserved mouth opening [29]. More recent work by Liu et al. also highlighted free flap success in over 130 pediatric patients undergoing H&N reconstruction, with careful attention to vessel size, age, and recipient site selection yielding reliable outcomes [30].

In our study, free flaps required longer operative and anesthesia times and were associated with increased hospitalization duration, reflecting their complexity and the perioperative demands of microsurgical reconstruction. However, the lower incidence of wound-related complications suggests that, in appropriately selected cases, free tissue transfer could offer a more durable solution for complex or irradiated defects [23,31,32,33]. Katsnelson et al. similarly reported that patients receiving pedicled myocutaneous flap reconstruction had significantly higher rates of surgical site infection, wound dehiscence, and readmission than patients receiving free tissue transfer, even after adjusting for comorbidities [34]. Moreover, with respect to donor site morbidity and mortality, a large pool of database evidence from ACS-NSQIP covering thousands of cases showed that while complication rates differ by flap type, neither flap category carried increased risk, as supported by our findings [35].

Our findings also emphasize that complications such as hardware exposure may result from the inability of certain local flaps to meet the structural demands of the defect. In this cohort, patients reconstructed with local flaps experienced higher rates of hardware exposure, particularly in irradiated fields or dynamic regions subject to growth and mechanical tension, such as the neck and mandible. In these anatomically demanding zones, pedicled tissue may lack the vascularity, bulk, or flexibility required to provide durable coverage over plates or alloplastic materials. This challenge is amplified in growing children, where soft tissue must accommodate skeletal expansion over time. When free tissue transfer is not feasible due to comorbidities, limited resources, or technical constraints, regional flaps may be considered. The trapezius flap, as described by Moshal et al., has demonstrated high survival rates and low donor site morbidity in posterior neck reconstruction in children [36]. However, its applicability for anterior defects is limited by its arc of rotation. For anterior neck reconstruction, pectoralis flaps remain a more commonly used alternative, but long-term data on their performance in pediatric oncologic patients, particularly regarding adaptability to growth, radiation effects, and hardware coverage, are lacking. Further studies are needed to evaluate the durability and functional outcomes of regional flaps in these complex settings.

Recent work by Youn et al. demonstrated that the use of custom endoprostheses in combination with free flaps significantly reduced hardware exposure (14.3% vs. 47.8%), complications, and unplanned revisions (11.1% vs. 50.0%) in pediatric mandibular reconstruction compared to stock reconstruction [37]. These findings underscore the value of pairing durable skeletal support with well-vascularized soft tissue coverage in high-risk reconstructions. Collectively, these data reinforce the importance of thoughtful flap selection and highlight the need for individualized, growth-conscious reconstructive strategies in children with oncologic defects.

In our institutional experience, some patients who underwent reconstruction with regional pedicled flaps ultimately required unplanned secondary procedures. The retrospective review of these cases suggests that primary free flap reconstruction might have preempted these adverse outcomes. This insight challenges the traditional reconstructive ladder paradigm, which emphasizes progressing from simpler to more complex options. In the context of pediatric head and neck oncology, where healing is impaired by chemoradiation, revisions pose a significant burden, and growth dynamics further complicate secondary interventions, a “reconstructive elevator” approach may be more appropriate. Proceeding directly to free tissue transfer in high-risk patients could reduce surgical morbidity and provide more definitive long-term reconstruction.

To aid reconstructive planning, we developed the “TARGETS” framework as a conceptual guide based on insights from this cohort. It is not intended as a rigid algorithm, but rather as a structured way to consider critical factors: Tissue type, Ability to heal, Radiation and restoration of function, Growth potential, Exposure, Tumor margins, and Site of resection (location) (Table 6). For example, in patients with prior radiation, free flaps provide healthy, well-vascularized tissue that can reduce wound healing complications [22,23,24]. When addressing growth, free flaps—particularly those involving bony reconstruction like the fibula—may better accommodate skeletal changes, as demonstrated in studies evaluating mandibular growth and function following fibular free flap reconstruction [38,39].

The high rate of major revisions observed across both cohorts (51.2%) underscores the complexity of reconstruction in pediatric oncologic patients. These children often undergo multimodal therapy, including chemotherapy and radiation, which impairs wound healing and immune function. These revision and wound dehiscence rates are likely multifactorial, influenced by the compounded effects of malignancy itself, chemoradiation, suboptimal nutritional status [25,40,41,42] and the extensive mean follow-up duration of two years, which captures late complications often missed in studies with shorter follow-ups. While the literature reports revision rates of up to 8% for head and neck reconstruction in children [36,37], our findings focus on a unique subset of pediatric patients with malignancies, underscoring the added complexity of their care. These results emphasize the need for tailored perioperative strategies to optimize outcomes in this high-risk population. In a retrospective series by Weizman et al., eight pediatric patients underwent free flap reconstruction following malignant head and neck tumor resection [20]. Although early and late complication rates were 50% and 25%, respectively, there were no flap losses. These findings reinforce the need for meticulous perioperative planning and long-term surveillance in this population.

Our study is not without limitations. This was a single-institution retrospective study with a relatively small sample size, limiting generalizability. Outcomes were not controlled by tumor stage, nutritional status, or radiation dose, factors that could influence wound healing and complication rates. Additionally, the heterogeneity in flap types and reconstructive indications limits direct comparisons. Nevertheless, this study provides one of the largest comparative analyses to date in a highly specific and understudied population.

## 5. Conclusions

In this retrospective comparison of free and local flap reconstruction in pediatric head and neck cancer patients, both techniques demonstrated high flap survival and comparable rates of major revisions. However, local flaps were associated with significantly higher rates of hardware exposure and wound dehiscence. These findings suggest that while both approaches are generally safe, free tissue transfer may be the preferred option in cases requiring long-term coverage over prosthetic material or in settings where soft tissue reliability is critical to avoid wound complications. We recommend prioritizing free flaps in irradiated or high-motion areas, reserving local flaps for smaller, superficial defects in stable regions. Microsurgical approaches are preferable when durable prosthetic coverage is needed, and both patient comorbidities and institutional capabilities should inform surgical planning. Finally, reconstruction strategies must account for anticipated growth and the long-term effects of radiation.

## Figures and Tables

**Table 1:** Demographics, tumor histological types, and preoperative treatment.

	All (*n* = 41)	Local Flaps (*n* = 23)	Free Flaps (*n* = 18)	*p*-Value
Age	10.6 ± 5.6	9.6 ± 5.5	11.9 ± 4.8	0.090
Sex				
Female	20 (48.8%)	11 (47.8%)	9 (50.0%)	0.749
Male	21 (51.2%)	12 (52.2%)	9 (50.0%)	
Pathology				
Osteosarcoma	9 (21.9%)	5 (21.7%)	4 (22.2%)	0.690
Spindle Cell Sarcoma	6 (14.6%)	4 (17.4%)	2 (11.1%)	
Rhabdomyosarcoma	5 (12.2%)	3 (13%)	2 (11.1%)	
Ewing Sarcoma	3 (7.3%)	1 (4.3%)	2 (11.1%)	
Clear Cell Sarcoma	1 (2.4%)	1 (4.3%)	-	
Epithelioid Sarcoma	1 (2.4%)	1 (4.3%)	-	
Synovial Sarcoma	1 (2.4%)	-	1 (5.6%)	
Fibrosarcoma	1 (2.4%)	1 (4.3%)	-	
Squamous Cell Carcinoma	2 (4.8%)	2 (8.7%)	-	
Malignant Giant Cell Tumor	1 (2.4%)	-	1 (5.6%)	
NUT Carcinoma	4 (9.8%)	2 (8.7%)	2 (11.1%)	
Medulloblastoma	5 (12.2%)	5 (21.7%)	-	
Neuroblastoma	1 (2.4%)	-	1 (5.6%)	
Preoperative Radiation				
Yes	27 (65%)	13 (56.5%)	14 (76.5%)	0.154
No	14 (34.1%)	10 (43.5%)	4 (22.2%)	

**Table 2:** Distribution of flap types for reconstruction.

Flap Type	Free Flaps (*n* = 18)
Anterolateral Thigh (ALT)	8 (44.4%)
Fibula	6 (33.3%)
Gracilis	3 (16%)
Rectus	1 (5.6%)
	Local/Regional Flaps (*n* = 23)
Facial	5 (21.7%)
Temporalis	5 (21.7%)
Trapezius	4 (17.4%)
Pectoralis	3 (13.0%)
Palatal	2 (8.7%)
Calvarial	1 (4.4%)
Cervicofacial	1 (4.4%)
Pericranial	1 (4.4%)
Platysma	1 (4.4%)

**Table 3:** Tissue transfer postoperative complications and revisions.

	All (*n* = 41)	Local Flaps (*n* = 23)	Free Flaps (*n* = 18)	*p*-Value
Flap Failure	1 (2.4%)	-	1 (5.5%)	0.252
Major Revisions	21 (51.2%)	13 (56.5%)	8 (44.4%)	0.443
Partial Flap Necrosis	6 (12.5%)	3 (13.0%)	3 (16.7%)	0.745
Hardware Exposure	9 (22.5%)	8 (34.7%)	1 (5.5%)	0.025 *
Wound Dehiscence	11 (25%)	9 (39.1%)	1 (5.5%)	0.013 *
Infection	7 (17.1%)	4 (17.4%)	3 (16.7%)	0.832
Hematoma	1 (2.4%)	1 (4.3%)	1 (5.5%)	0.859

Significant values (*p* < 0.05) are in bold and with a (*).

**Table 4:** Operative times, LOH, readmission, follow-up.

	All (*n* = 41)	Local Flaps (*n* = 23)	Free Flaps (*n* = 18)	*p*-Value
Operative Time (Hours)	8.2 ± 5.1	6.5 ± 1.2	10.3 ± 0.8	0.011 *
Anesthesia Duration (Hours)	9.5 ± 5.7	7.9 ± 5.9	12.5 ± 4.1	0.015 *
Length of Hospitalization (days)	20.9 ± 25.6	13.9 ± 3.5	29.1 ± 7.9	0.036 *
Readmission within 30 days	13 (32.5%)	10 (43.5%)	3 (17.6%)	0.085
Follow-up (Years)	2.3 ± 2.9	2.7 ± 3.8	1.8 ± 1.8	0.788

Significant values (*p* < 0.05) are in bold and with a (*).

**Table 5:** Mortality and donor site morbidity.

	All (*n* = 41)	Local Flaps (*n* = 23)	Free Flaps (*n* = 18)	*p*-Value
Donor Site Morbidity	1 (2.4%)	-	1 (5.6%)	0.252
Total Flap Reoperations	1.4 ± 1.9	1.7 ± 2.2	1.1 ± 1.7	0.822
Mortality	14 (26.8%)	6 (26.1%)	5 (27.8%)	0.903
Age at Mortality	13.5 ± 7.1	9.8 ± 9.9	15.6 ± 4.9	0.148
Time from Tissue Transfer to Mortality	1.9 ± 1.8	1.2 ± 0.6	2.3 ± 1.0	0.235

**Table 6:** Comparative considerations for local versus free tissue transfer in pediatric H&N reconstruction.

TARGETS	Local Tissue Transfer	Free Tissue Transfer
Type of tissue	Suitable for soft tissue-only defects.	Preferred for composite tissue requirements (bone, muscle, skin).
Ability to heal	Local flaps may be more prone to wound dehiscence, especially in irradiated or thin tissue beds.	Free flaps provide well-vascularized tissue that may reduce the risk of dehiscence and promote more reliable healing.
Restoration of function/ radiation	Adequate for simpler functional restorations. Radiation might affect regional tissue quality and vascularity, making local tissue transfer less optimal.	Superior for restoring complex functions like speech or chewing. Offers well-vascularized, non-irradiated tissue for repair.
Growth potential	May require additional surgical revisions and adjustments as the child grows.	Greater recruitment of soft and bone tissue may better accommodate the child’s growth over time.
Exposure	May offer adequate soft tissue coverage for hardware. May need additional revisions for hardware exposure.	May offer greater recruitment of soft tissue for hardware long-term coverage.
Tumor margins	May not adequately address extensive margins. Sufficient for small defects.	Allows for wide reconstruction with extensive margins. Preferred for larger defects.
Site of resection	Limited to regional tissue near the defect; may not suffice for extensive or deep defects.	Enables reconstruction across distant or complex anatomical sites where local tissue is insufficient.

The table outlines critical factors using the mnemonic “TARGETS,” which include type of tissue, ability to heal, restoration of function and radiation impact, growth potential, exposure, tumor margins, and site (location) of resection.

## Data Availability

The data presented in this study are not publicly available due to ethical and institutional restrictions. The dataset contains sensitive patient information collected under IRB approval and cannot be shared to protect patient privacy and comply with HIPAA regulations.

## Abbreviations

The following abbreviations are used in this manuscript:

PRS	Plastic and Reconstructive Surgery
CSF	Cerebrospinal Fluid
CHLA	Children’s Hospital Los Angeles
H&N	Head and Neck
ALT	Anterolateral Thigh

## References

[1] Albright J.T., Topham A.K., Reilly J.S. (2002). Pediatric Head and Neck Malignancies: US Incidence and Trends Over 2 Decades. Arch. Otolaryngol. Head Neck Surg..

[2] Van Landuyt K., Hamdi M., Blondeel P., Tonnard P., Verpaele A., Monstrey S. (2005). Free perforator flaps in children. Plast. Reconstr. Surg..

[3] Chim H., Salgado C.J., Seselgyte R., Wei F.C., Mardini S. (2010). Principles of head and neck reconstruction: An algorithm to guide flap selection. Semin. Plast. Surg..

[4] Hanasono M.M., Matros E., Disa J.J. (2014). Important aspects of head and neck reconstruction. Plast. Reconstr. Surg..

[5] Ragbir M., Brown J.S., Mehanna H. (2016). Reconstructive considerations in head and neck surgical oncology: United Kingdom National Multidisciplinary Guidelines. J. Laryngol. Otol..

[6] Kim H.S., Chung C.H., Chang Y.J. (2020). Free-flap reconstruction in recurrent head and neck cancer: A retrospective review of 124 cases. Arch. Craniofac. Surg..

[7] Eckardt A., Fokas K. (2003). Microsurgical reconstruction in the head and neck region: An 18-year experience with 500 consecutive cases. J. Craniomaxillofac. Surg..

[8] Amarante J., Reis J., Costa-Ferreira A., Malheiro E., Silva A. (2000). Head and neck reconstruction: A review of 117 cases. Eur. J. Plast. Surg..

[9] Cordeiro P.G., Disa J.J., Hidalgo D.A., Hu Q.Y. (1999). Reconstruction of the mandible with osseous free flaps: A 10-year experience with 150 consecutive patients. Plast. Reconstr. Surg..

[10] Dorji K. (2024). Local or regional flaps in developing country: Experience from Eastern Bhutan. Int. Wound J..

[11] Upton J., Guo L. (2008). Pediatric free tissue transfer: A 29-year experience with 433 transfers. Plast. Reconstr. Surg..

[12] Konttila E., Koljonen V., Kauhanen S., Kallio P., Tukiainen E. (2010). Microvascular reconstruction in children: A report of 46 cases. J. Trauma.

[13] Dormand E.L., Banwell P.E., Goodacre T.E. (2005). Radiotherapy and wound healing. Int. Wound J..

[14] Robinson M.D., McNamara M.G., Clouston H.W., Sutton P.A., Hubner R.A., Valle J.W. (2023). Patients Undergoing Systemic Anti-Cancer Therapy Who Require Surgical Intervention: What Surgeons Need to Know. Cancers.

[15] Arnold D.J., Wax M.K. (2007). Microvascular Committee of the American Academy of Otolaryngology-Head and Neck Surgery. Pediatric microvascular reconstruction: A report from the Microvascular Committee. Otolaryngol. Head Neck Surg..

[16] Warren S.M., Borud L.J., Brecht L.E., Longaker M.T., Siebert J.W. (2007). Microvascular reconstruction of the pediatric mandible. Plast. Reconstr. Surg..

[17] Yazar S., Wei F.C., Cheng M.H., Huang W.C., Chwei-Chin Chuang D., Lin C.H. (2008). Safety and reliability of microsurgical free tissue transfers in paediatric head and neck reconstruction—A report of 72 cases. J. Plast. Reconstr. Aesthet. Surg..

[18] Jacob L.M., Dong W., Chang D.W. (2010). Outcomes of reconstructive surgery in pediatric oncology patients: Review of 10-year experience. Ann. Surg. Oncol..

[19] Zhang C., Sun J., Zhu H., Xu L., Ji T., He Y., Yang W., Hu Y., Yang X., Zhang Z. (2015). Microsurgical free flap reconstructions of the head and neck region: Shanghai experience of 34 years and 4640 flaps. Int. J. Oral Maxillofac. Surg..

[20] Weizman N., Gil Z., Wasserzug O., Amir A., Gur E., Margalit N., Fliss D.M. (2011). Surgical ablation and free flap reconstruction in children with malignant head and neck tumors. Skull Base.

[21] Nakatsuka T., Harii K., Yamada A., Yonehara Y., Takato T., Kawahara N., Sasaki T., Yamasoba T., Nibu K., Ebihara S. (1998). Immediate free flap reconstruction for head and neck pediatric malignancies. Ann. Plast. Surg..

[22] Aboelatta Y.A., Aly H.M. (2013). Free tissue transfer and replantation in pediatric patients: Technical feasibility and outcome in a series of 28 patients. J. Hand Microsurg..

[23] Nepon H., Safran T., Reece E.M., Murphy A.M., Vorstenbosch J., Davison P.G. (2021). Radiation-Induced Tissue Damage: Clinical Consequences and Current Treatment Options. Semin. Plast. Surg..

[24] Martinovic D., Tokic D., Puizina Mladinic E., Usljebrka M., Kadic S., Lesin A., Vilovic M., Lupi-Ferandin S., Ercegovic S., Kumric M. (2023). Nutritional Management of Patients with Head and Neck Cancer-A Comprehensive Review. Nutrients.

[25] Gorenc M., Kozjek N.R., Strojan P. (2015). Malnutrition and cachexia in patients with head and neck cancer treated with (chemo)radiotherapy. Rep. Pract. Oncol. Radiother..

[26] Jan W.L., Chen H.C., Chang C.C., Chen H.H., Shih P.K., Huang T.C. (2020). Modified Clavien-Dindo Classification and Outcome Prediction in Free Flap Reconstruction among Patients with Head and Neck Cancer. J. Clin. Med..

[27] Starnes-Roubaud M.J., Hanasono M.M., Kupferman M.E., Liu J., Chang E.I. (2017). Microsurgical Reconstruction Following Oncologic Resection in Pediatric Patients: A 15-Year Experience. Ann. Surg. Oncol..

[28] Akçal A., Karşıdağ S., Sucu D.Ö., Turgut G., Uğurlu K. (2013). Microsurgical reconstruction in pediatric patients: A series of 30 patients. Ulus. Travma Acil Cerrahi Derg..

[29] Zavala A., Ore J.F., Broggi A., De Pawlikowski W. (2021). Pediatric Mandibular Reconstruction Using the Vascularized Fibula Free Flap: Functional Outcomes in 34 Consecutive Patients. Ann. Plast. Surg..

[30] Liu S., Zhang W.B., Wang Y., Mao C., Yu G.Y., Peng X. (2024). Long-Term Outcomes After Pediatric Mandibular Reconstruction Using Vascularized Free Fibula Flap. Plast. Reconstr. Surg..

[31] Parker R.J., Eley K.A., Von Kier S., Pearson O., Watt-Smith S.R. (2012). Functional fibrinogen to platelet ratio using thromboelastography as a predictive parameter for thrombotic complications following free tissue transfer surgery: A preliminary study. Microsurgery.

[32] Rosen R.D., Manna B. (2024). Wound Dehiscence. StatPearls.

[33] Deptuła M., Zieliński J., Wardowska A., Pikuła M. (2019). Wound healing complications in oncological patients: Perspectives for cellular therapy. Postepy Dermatol. Alergol..

[34] Katsnelson J.Y., Tyrell R., Karadsheh M.J., Manstein E., Egleston B., Deng M., Baltodano P.A., Shafqat M.S., Patel S.A. (2022). Postoperative Complications Associated with the Choice of Reconstruction in Head and Neck Cancer: An Outcome Analysis of 4712 Patients from the ACS-NSQIP Database. J. Reconstr. Microsurg..

[35] Mahieu R., Colletti G., Bonomo P., Parrinello G., Iavarone A., Dolivet G., Livi L., Deganello A. (2016). Head and neck reconstruction with pedicled flaps in the free flap era. Acta Otorhinolaryngol. Ital..

[36] Moshal T., Lasky S., Roohani I., Jolibois M.I., Manasyan A., Munabi N.C.O., Fahradyan A., Lee J.A., Hammoudeh J.A. (2025). The Forgotten Flap: The Pedicled Trapezius Flap’s Utility in Pediatric Head and Neck Reconstruction—A Systematic Review. J. Reconstr. Microsurg..

[37] Youn S., Trotter C., O’Brien D., Alfeerawi S., Choi D., Roohani I., Shakoori P., Fahradyan A., Urata M.M., Hammoudeh J.A. (2023). A Novel Algorithm for Pediatric Microsurgical Maxillomandibular Reconstruction Using Custom Endoprosthesis. J. Oral Maxillofac. Surg..

[38] Liu S., Zhang W.B., Yu Y., Wang Y., Mao C., Guo C.B., Yu G.Y., Peng X. (2019). Free Flap Transfer for Pediatric Head and Neck Reconstruction: What Factors Influence Flap Survival?. Laryngoscope.

[39] Zhang W.B., Liang T., Peng X. (2016). Mandibular growth after paediatric mandibular reconstruction with the vascularized free fibula flap: A systematic review. Int. J. Oral Maxillofac. Surg..

[40] Gil Z., Patel S.G., Singh B., Cantu G., Fliss D.M., Kowalski L.P., Kraus D.H., Snyderman C., Shah J.P., International Collaborative Study Group (2007). Analysis of prognostic factors in 146 patients with anterior skull base sarcoma: An international collaborative study. Cancer.

[41] Sunkara P.R., Graff J.T., Cramer J.D. (2023). Association of Surgical Margin Distance with Survival in Patients with Resected Head and Neck Squamous Cell Carcinoma: A Secondary Analysis of a Randomized Clinical Trial. Arch. Otolaryngol.—Head Neck Surg..

[42] Allam O., Shah R., Cadwell J.B., Dinis J., Peck C., Junn A., Gowda A., Alperovich M. (2022). Evaluation of Complication Rates of Free Flap Reconstruction in Pediatric Patients. J. Indian Assoc. Pediatr. Surg..

